# Hemoadsorption in ‘Liver Indication’—Analysis of 109 Patients’ Data from the CytoSorb International Registry

**DOI:** 10.3390/jcm10215182

**Published:** 2021-11-05

**Authors:** Klementina Ocskay, Dana Tomescu, Andreas Faltlhauser, David Jacob, Sigrun Friesecke, Manu Malbrain, Klaus Kogelmann, Ralph Bogdanski, Friedhelm Bach, Harald Fritz, Andreas Hartjes, Andreas Kortgen, Jens Soukup, Stefan Utzolino, Martijn van Tellingen, Karl Träger, Ulrike Schumacher, Frank M. Brunkhorst, Zsolt Molnar

**Affiliations:** 1 Institute for Translational Medicine, Medical School, University of Pécs, 7624 Pécs, Hungary; ocskay.klementina@gmail.com; 2 Anesthesiology and Intensive Care, Fundeni Clinical Institute, 022328 Bucharest, Romania; danatomescu@gmail.com; 3 Medizinische Klinik I, Kliniken Nordoberpfalz AG, Klinikum Weiden, 92637 Weiden, Germany; andreas.faltlhauser@kliniken-nordoberpfalz.ag; 4 Klinik für Anaesthesiologie und Intensivtherapie, Universitätsklinikum Magdeburg, 39120 Magdeburg, Germany; david.jacob@med.ovgu.de; 5 Klinik und Poliklinik für Innere Medizin B, Universitätsmedizin Greifswald, 17475 Greifswald, Germany; sigrun.friesecke@med.uni-greifswald.de; 6 Intensive Care Chief Medical Officer, AZ Jan Palfijn, 9000 Gent, Belgium; manu.malbrain@janpalfijngent.be; 7 First Department of Anaesthesia and Intensive Therapyx, Medical University of Lublin, 20059 Lublin, Poland; 8 Hans-Susemihl-Krankenhaus GmbH, Klinik für Anästhesiologie und Intensivmedizin, 26721 Emden, Germany; k.kogelmann@klinikum-emden.de; 9 Klinikum Rechts der Isar der TU München, Klinik für Anästhesiologie, 81675 München, Germany; ralph.bogdanski@tum.de; 10 Evangelisches Krankenhaus Bielefeld, Klinik für Anästhesiologie, Intensiv-, Notfallmedizin, Transfusionsmedizin und Schmerztherapie, Kantensiek 11, 33617 Bielefeld, Germany; friedhelm.bach@evkb.de; 11 Krankenhaus Martha Maria Halle, Klinik für Anaesthesiologie und Intensivmedizin, Röntgenstr. 1, 06120 Halle, Germany; harald.fritz@martha-maria.de; 12 Krankenhaus der Barmherzigen Schwestern Ried, Anästhesie, Intensiv- und Palliativmedizin, Schlossberg 1, 4933 Ried im Innkreis, Austria; andreas.hartjes@bhs.at; 13 Klinik für Anästhesiologie und Intensivmedizin, Universitätsklinikum Jena, 07747 Jena, Germany; andreas.kortgen@med.uni-jena.de; 14 Carl-Thiem-Klinikum Cottbus, Klinik für Anästhesiologie, Intensivtherapie und Palliativmedizin, Thiemstr. 111, 03048 Cottbus, Germany; j.soukup@ctk.de; 15 Abteilung Allgemein- und Viszeralchirurgie, Universitätsklinikum Freiburg, 79106 Freiburg, Germany; stefan.utzolino@uniklinik-freiburg.de; 16 Intensive Care, Ziekenhuis de Tjongerschans, 8441 PW Heerenveen, The Netherlands; martijn.van.tellingen@tjongerschans.nl; 17 Kardioanasthesiologie, Universitätsklinikum Ulm, Albert-Einstein-Allee 23, 89081 Ulm, Germany; karl.traeger@uni-ulm.de; 18 Zentrum für Klinische Studien, Universitätsklinikum Jena, 07747 Jena, Germany; ulrike.schumacher@med.uni-jena.de; 19 Zentrum für Klinische Studien, Klinik für Anästhesiologie und Intensivtherapie, Universitätsklinikum Jena, Am Klinikum 1, 07747 Jena, Germany; frank.brunkhorst@med.uni-jena.de; 20 Department of Anaesthesiology and Intensive Therapy, Faculty of Medicine, Poznan University for Medical Sciences, 60355 Poznan, Poland; 21 Department of Anaesthesiology and Intensive Therapy, Semmelweis University, 1082 Budapest, Hungary

**Keywords:** CytoSorb, cytokine adsorption, hemoadsorption, liver failure, bilirubin, liver support, blood purification

## Abstract

Background: Our aim is to report the results of the ‘liver indication’ subset of patients in the CytoSorb International Registry. Methods: Structured data were recorded. Treatment characteristics and changes from T1 (start of hemoadsorption) to T2 (termination) were evaluated with a special focus on bilirubin, C-reactive protein, procalcitonin, interleukin-6, platelet levels, SOFA scores, mortality, and subjective assessment by the attending physicians. Results: Until January 2021, from the total 1434 patients, 109 (age: 49.2 ± 17.1 years, 57.8% males) received treatment for hyperbilirubinemia. APACHE II-predicted mortality was 49.6 ± 26.8%. In the study, 91% of patients were alive at the termination of hemoadsorption and improvement was observed by the physicians in 75 cases. Overall, 65 (59.6%) patients died in the hospital, and 60 (55.0%) died in the ICU. Patients received a median of two treatments for a median of 43 h (interquartile range: 24–72 h) in total. Serum bilirubin levels reduced significantly to −4.6 (95% CI: −6.329 to −2.8) mg/dL. Thrombocytopenia was reported in four patients as an adverse event. Conclusions: We report the largest case series on hemoadsorption for ‘liver indication’ from the CytoSorb International Registry. The finding of significant bilirubin removal observed in our study could have substantial impact in designing and executing further studies on the effects of hemoadsorption in liver dysfunction, which are certainly warranted.

## 1. Introduction

Dysregulated host response, initiated by infectious or non-infectious injuries, can lead to multiple organ dysfunction [1,2]. Up to 20% of the critically ill patients develop liver injury during their course of critical illness. Several scores have been developed for the assessment of liver dysfunction with the common feature that all include serum bilirubin levels [3,4]. It is generally accepted that monitoring of the excretory function of the liver is essential in critically ill patients, for which bilirubin is the appropriate marker [5]. There is also significant correlation between serum bilirubin levels and mortality in several critically ill conditions [6,7,8].

CytoSorb (CytoSorbents, Monmouth Junction, NJ, USA) is a relatively new extracorporeal blood purification device. It is a 300 mL container filled with biocompatible, highly porous polystyrene divinylbenzene beads that form a large surface of about 45,000 m^2^, adsorbing hydrophobic molecules up to approximately 55 kDa. As most cytokines fall within this range, the device is potentially capable of eliminating toxic substances rapidly from the blood. CytoSorb was primarily used in septic shock and other hyperinflammatory conditions for removing inflammatory mediators and hence preventing the harmful effects of cytokine storm as a result of an overwhelming inflammatory response [9]. The device was registered in 2011 and the number of patients treated with CytoSorb is continuously growing, so far more than 152,000 treatments in various indication fields were carried out worldwide (information from CytoSorbents, https://cytosorb-therapy.com/en/the-therapy/ (accessed on 28 September 2021).

In May 2015, the CytoSorb International Registry was established independently by the Center for Clinical Studies at the Jena University Hospital, Germany. The number of centers participating and the number of patients included increased since the first paper on the 3rd interim analysis of 198 patients’ data was published in 2017 [10], until it was closed in November 2020. Its aim was to collect data on the clinical practice on the use of hemoadsorption in intensive care patients.

Extracorporeal hemoadsorption is used by intensive care specialists mostly to prevent or counterbalance the effects of cytokine storm mainly in septic shock and other forms of vasoplegic shock [11]. Acute forms of liver dysfunction and failure also share the pathophysiological features of dysregulated inflammatory response with sepsis, which serves as the basis of the rationale hemoadsorption in liver dysfunction [12,13,14]. There are some case reports, case series, and in vitro data indicating effective removal of bilirubin, bile acids, and ammonia from the blood by using hemoadsorption [15,16,17,18]. In a recent in vitro study, CytoSorb was also found superior to albumin dialysis (Molecular Adsorbent Recirculating System: MARS) as far as bilirubin, bile acid, ammonia, and cytokine removal are concerned [17]. However, large prospective data or results of randomized trials are still missing. Nevertheless, in the Registry, the entries on treatment with CytoSorb for ‘liver indication’ have also increased over the last few years.

Therefore, we felt it would be relevant to analyze this subgroup of the CytoSorb International Registry separately and disseminate the results in the medical community.

## 2. Materials and Methods

### 2.1. Enrollment

Participation in the International CytoSorb Registry was open for every center where CystoSorb treatment is used. After registration at www.cytosorb-registry.org (accessed on 28 September 2021) and ethical approval, data were collected and uploaded into the database. Any adult patient (>18 years) who received CytoSorb treatment was eligible after informed consent was acquired from the patient or close relatives (if the patient is unable to provide consent, according to local regulations). No exclusion criteria were specified.

### 2.2. Patients

In the Registry, enrolled patients were categorized by indication of CytoSorb therapy. The following four categories were defined:

- Sepsis/septic shock, *n* = 936;
- CytoSorb use during cardiac surgery (preemptive), *n* = 172;
- CytoSorb use after cardiac surgery (postoperative), *n* = 67;
- Other indications, *n* = 259.

In the ‘other indication’ group, the biggest subgroup was thmose receiving CytoSorb therapy for ‘liver indication’ (*n* = 109).

### 2.3. Interventions

There were no specific interventions apart from CytoSorb therapy that was recommended to be used according to the user’s guide provided by CytoSorbents. The cartridge needs to be incorporated into an extracorporeal circuit and used on its own as hemoperfusion, or together with renal replacement therapy. One adsorber is recommended to be used for up to 24 h.

### 2.4. Data Collection and Management

The International CytoSorb Registry was registered at ClinicalTrials.gov (ClinicalTrials.gov, accessed on 28 September 2021, Identifier: NCT02312024) and the German Registry for Clinical Studies Freiburg (DRKS). Electronic case report forms were used. Data were recorded at baseline, up to 24 h before treatment (T1), up to 24 h after the last CytoSorb therapy (T2) and at discharge from the hospital or mortality. Data collection was performed by dedicated staff, who were trained by the CytoSorb Registry Project Manager. All data were handled confidentially and stored on the servers of the Center for Clinical Studies at Jena University Hospital using the OpenClinica study management software. A detailed description of the data collection and management has been discussed previously [10].

### 2.5. Outcomes

The main outcome parameters evaluated by the Registry were the following: in-hospital mortality, mortality at the end of CytoSorb therapy, and ICU mortality as compared to the APACHE II-predicted mortality; length of hospital stay (given in days), duration of mechanical ventilation (MV, days), duration of renal replacement therapy (RRT, days), and duration of vasopressor therapy (days); changes in total bilirubin, C-reactive protein (CRP), procalcitonin (PCT), interleukin-6 (IL-6), and platelet levels; sequential organ failure assessment (SOFA) score; and change of the patient’s condition. Whether patients’ general condition improved, did not change, or got worse during and after hemoadsorption was also assessed by the attending physician, using a subjective scale.

### 2.6. Statistics

During the final analysis of the CytoSorb International Registry, a separate analysis was performed using the dataset of the patients for whom the indication of therapy was labelled as ‘liver’ dysfunction. Descriptive statistical methods were used. The count and percentage were provided for ordinal outcomes and categories. For continuous outcomes, number (*n*), mean, median, interquartile (Q3–Q1), standard deviation, minimum, and maximum values are displayed. Changes were calculated from T1 and T2 values using a *t*-test as well as a linear model for selected variables. Additionally, 95% confidence intervals (CI) were provided to indicate the size of the observed effect.

## 3. Results

From 18 May 2015 until 29 January 2021, 1434 patients were entered into the Registry from nine countries by 46 centers. The indication for CytoSorb treatment was sepsis/septic shock in 936, pre-emptive use during cardiac surgery in 172, and postoperative use after cardiac surgery in 67 cases, and “other” reasons in 259 cases. The overall results of the Registry will be published elsewhere. Out of the study group, 109 patients were entered into the ‘liver’ subgroup. Patients were enrolled in five countries (Austria, Belgium, Germany, the Netherlands, and Romania) at 17 centers. Data were available at T1 for 109 and at T2 for 107 patients. The mean age was 49.2 years, and the majority of patients were male (57.8%). Comorbidities were absent in almost half of the patients, but some sort of liver dysfunction was also present in a substantial number of patients (Table 1). Further baseline characteristics are depicted in Table 1.

Patients received a median of 2 treatments (i.e., 2 adsorbers), 43 patients were treated once only, and the maximum number of treatments a patient received was 25. The overall duration of treatment was a median of 46 h. The median time of use per adsorber was 24 h and time between treatments was less than 1 h (median) (Table 2).

More than 90% of patients were alive at the termination of CytoSorb therapy (Table 3). Overall, 60 patients died in the ICU and 5 more during hospitalization, which corresponds to a 59.6% in-hospital mortality. Non-survivors required mechanical ventilation, RRT, and vasopressor support for longer as compared to survivors, but spent a shorter time in the ICU (median 11 vs. 16 days). As far as SOFA scores are concerned, only the coagulation subscore changed significantly from 24 h before and 24 h after the course of hemoadsorption, caused by a drop in platelet count. There was an improvement in the SOFA liver subscore (based on bilirubin levels), but this did not reach significance. The mean SOFA CNS subscore was 1.3 at T1, corresponding to a Glasgow Coma Score (GCS) of around 12–14, which did not change significantly after treatment (Table 3).

The most profound change from T1 to T2 was observed in the serum bilirubin levels (Table 3).

Inflammatory markers did not change significantly over time in general. IL-6 was measured only in 13 patients at T1 and only in 7 patients at T2; therefore, changes could not be evaluated (Table 3).

The attending physicians observed improvement in 75 cases (68.9%), no change was registered in 17 cases (15.6%), and a worsening condition was reported in 5 (4.8%) patients (Table 3).

Regarding safety, device-related adverse events were reported in four patients. Thrombocytopenia was observed in all four cases, without any bleeding complications, and in one patient concomitant hyponatremia and the decrease of kidney function was reported.

## 4. Discussion

In the current article we report the results of the largest case series of CytoSorb treatment in critically ill patients who received hemoadsorption for ‘liver indication’. Although the Registry was not designed to collect specific data on acute or acute-on-chronic liver failure, because at the time of its construction (in 2014), this indication was unexpected, we believe that the results of our study could have significant impact on both daily clinical practice (i.e., considering hemoadsorption in critically ill patients with acute onset liver dysfunction that is not improving for standard medical therapy) and also in designing and executing future clinical trials that are certainly needed. This notion is further emphasized by the fact that, without any supporting evidence, the ‘liver indication’ turned out to be the third most frequent indication in the Registry, making up 7.6% of all cases.

According to the comorbidities listed in Table 1, a substantial number of patients suffered from one or several liver-related conditions, but it is also worth noting that almost 40% of patients were free of comorbidities; hence, ‘liver indication’ was most likely established within the context of multiple organ dysfunction. Unfortunately, based on the lack of detailed data, we cannot make further assessment in this regard. In general, removal of toxins that accumulate during liver failure from the circulation is the main aim during extracorporeal liver support in general. There are several treatment modalities; however, clear evidence about the best method cannot be determined as we recently confirmed it in two recent meta-analyses in both acute and acute-on-chronic liver failure [19,20], and we proposed the need for both good quality randomized studies and testing new alternatives. In our view, uncertainties about the benefits of currently available liver support therapies have encouraged several centers to try new blood purification techniques in liver dysfunction. This is further supported by the results of the Registry.

Our results show that hemoadsorption effectively removes bilirubin from the blood, which has only been shown by a few studies with small sample size or on retrospectively collected data [21,22,23]. The mean serum bilirubin level was 12 mg/dL, which corresponds to the highest score (+4) in the SOFA score system, indicating that, in most patients, hyperbilirubinemia was present. Although reduction of bilirubin levels has also been reported during the currently used extracorporeal liver support therapies, different devices have different removal rates [24]. Additionally, according to a very recent in vitro study comparing MARS to CytoSorb hemoadsorption, the latter removes tumor necrosis factor-α, IL-6, and ammonia, as well as albumin-bound toxins such as indirect bilirubin and bile acids more effectively than MARS [17].

Nevertheless, the buildup of toxic substances is not only due to the deterioration of hepatic functions, but may also be due to renal impairment, something that is often seen in multiple system organ failure and also in liver failure. Acute kidney injury develops in about 60% of the critically ill [25] and in 40% in liver failure [26,27,28]. Furthermore, the accumulation of bilirubin in the distal nephron segments and proximal tubules in the kidneys contributes to kidney injury often seen in liver failure and the critically ill [29]. Therefore, it is not surprising that 98% of adsorbers were administered together with continuous renal replacement therapy. However, it is important to note that the indication why CRRT was commenced cannot be confirmed from the Registry data, neither can we tell how many patients required renal support for acute renal failure. In fact, mean serum creatinine and HCO3 levels were only mildly elevated (creatinine of 1.8 mg/dL corresponds to a SOFA subscore of +1), and unfortunately, we have no data on urinary output. Therefore, one cannot exclude that, in several instances, hemoadsorption was coupled with CRRT for reimbursement or local regulatory reasons, something that has to be investigated further in the future.

Regarding survival, more than 90% of patients were still alive at the end of hemoadsorption therapy and improvement was observed by the treating physicians in almost 70% of cases. Patients received a median of only two treatments, after which physicians terminated hemoadsorption with a relatively high satisfaction rate, one could assume that the main reason for stopping the therapy was overall improvement. Unfortunately, objective proof of this assumption—apart from the reduction of bilirubin levels—cannot be retrieved from the Registry data. Both ICU and hospital mortality was higher than predicted by admission APACHE-II scores; nevertheless, patients stayed in the ICU for about 2 weeks after hemoadsorption was terminated (regardless of whether they survived or not), which makes us postulate that deaths occurred due to reasons other than what hemoadsorption was started for. It is also important to note that in future studies/registries especially designed for patients treated due to liver dysfunction, liver-specific scores should be used instead of APACHE-II, such as the CLIF-SOFA or the LiFe scores [30,31].

Although platelet count decreased significantly, but this is a frequent finding in patients receiving continuous renal replacement therapy and according to Lisman et al., thrombocytopenia is of little clinical significance in liver failure [32]. Nevertheless, it is difficult to know whether this drop in platelet count was due to the hemoadsorption therapy or to the extracorporeal treatment itself. In the current study, in one case clotting of the cartridge was reported during therapy, and only in four cases were thrombocytopenia as adverse events reported, without any bleeding complications. However, it is important to note that the registry is unable to answer all issues concerning safety at the moment, including changes in platelet count, removal of certain drugs, etc. These are questions that must be answered in future randomized trials.

### Strengths and Limitations

This is the largest dataset in this topic so far, for which systematic data were collected in a prospective, multicenter fashion. One of the main limitations is that the Registry was not designed specifically for patients with liver dysfunction/failure; hence, on the one hand, the entry of the patients into ‘liver indication’ group was at the discretion of the attending physician without any preset specification and, on the other hand, we cannot make any definitive comment on the role of CytoSorb therapy in liver failure in general, apart from the observation that bilirubin levels significantly reduced during the therapy. Nor can we comment on timing, neither on the pathophysiological reasons why hemoadsorption was actually started, as data were not collected. There were also several missing data, which was mainly due to the fact that sites did not receive any reimbursement or financial support to employ dedicated personnel for this task. Therefore, these limitations can serve as a useful basis when designing future registries on hemoadsorption. Furthermore, we have no control group either to compare these results. Data on ammonia levels, bile acids, and transplantation were not recorded either.

Therapeutic decisions were guided by local protocols, or simply by the physicians’ intuition; therefore, this work serves best for hypothesis generation. To answer specific therapy-related questions, randomized controlled trials are needed.

## 5. Conclusions

This paper summarizes the results of the ‘liver indication’ subset of patients from the CytoSorb International Registry, which turned out to be the third largest cohort after sepsis/septic shock and cardiac surgery. The finding that significant bilirubin removal took place during the therapy is encouraging, but further studies are required to elaborate the effects of hemoadsorption in liver dysfunction and/or failure. We believe that even until these randomized trials are completed, the current results could have substantial impact in both application of hemoadsorption in daily practice and also in designing appropriate clinical trials that provide evidence on the use of hemoadsorption in acute liver dysfunction. 

## Figures and Tables

**Table 1:** Demographics, baseline parameters.

Parameter	Mean ± SD ^4^; Median [IQR] ^5^	N
Age (years)	49.2 ± 17.1	109
Male/Female	63/46	109
APACHE II score	20.3 ± 9.5	89
Predicted mortality (%)	49.6 ± 26.8	89
Comorbidities ^1^:		
none	-	43 (39.4)
biopsy confirmed cirrhosis	-	33 (30.3)
confirmed portal hypertension	-	37 (33.9)
past upper gastrointestinal bleeding	-	18 (16.5)
previous liver failure	-	19 (17.0)
previous hepatic encephalopathy	-	16 (14.7)
other (cardiorespiratory, malignancy)	-	48 (44.0)
SOFA ^2^ score T1	12.1 ± 4.3	89
SOFA CNS ^3^ subscore	1.3 ± 1.5	106
SOFA cardiovascular subscore	1.9 ± 1.7	106
SOFA pulmonary subscore	2.0 ± 1.6	97
SOFA coagulation subscore	1.8 ± 1.2	108
SOFA liver subscore	3.1 ± 1.2	108
SOFA renal subscore	2.1 ± 1.6	108
Serum bicarbonate (mmol/L)	19.8 ± 4.7	103
Serum sodium (mmol/L)	134.9 ± 8.0	108
Serum potassium (mmol/L)	3.9 ± 0.6	108
Serum creatinine (mg/dL)	1.8 ± 1.6	104
Serum urea (pg/mL)	14.6 ± 11.5	105
Serum total bilirubin (mg/dL)	12.0 (3.9–24.7)	108
Leukocytes (G/L)	12.1 ± 7.2	107
Platelets (G/L)	98.1 ± 69.4	108
C-reactive protein (mg/L)	56.4 ± 68.8; 30.1 (14.0–81.9)	85
Procalcitonin (ng/mL)	3.9 ± 6.4; 1.1 (0.6–4.3)	64
Interleukine-6 (pg/mL)	136,558.5 ± 492,350.5; 149.9 (69.2–969.9)	13

^1^ Number of patients with the registered comorbidity; ^2^ Sequential Organ Failure (SOFA) subscores range from 0 to 4 with an increasing score reflecting worsening of organ dysfunction; ^3^ CNS: central nervous system; ^4^ SD: standard deviation; ^5^ IQR: interquartile range.

**Table 2:** Treatment characteristics.

Parameter		N
Number of adsorbers (median [IQR] ^1^)	2 (1–4)	109 ^2^
Total duration of treatment (h) (median [IQR])	46 (24–72.3)	109 ^2^
Treatment time per adsorber (h) (median [IQR])	24 (15.3–24.0)	319 ^3^
Time between treatments (h) (median [IQR])	0.2 (0.0–10.2)	210 ^4^
Blood pump flow rate (mL/min) (median [IQR])	140 (100–160)	319 ^3^
Combined with renal replacement therapy, *n* (%)	309 (97.8%)	316 ^3^

^1^ IQR: interquartile range; ^2^ number of patients; ^3^ number of adsorbers; ^4^ number of events.

**Table 3:** Outcome parameters.

Parameter		N
Mortality at the end of hemoadsorption, *n* (%)	10 (9.2)	109
ICU ^1^ mortality, *n* (%)	60 (55.0)	109
Hospital mortality, *n* (%)	65 (59.6)	109
ICU length of stay (days, median (IQR) ^4^)	14.0 (7.0–23.0)	107
ICU length of stay—survivors (days, median (IQR))	16.0 (8.0–26.0)	49
ICU length of stay—non-survivors (days, median (IQR))	11.0 (7.0–21.0)	58
Mechanical ventilation (days, median (IQR))	5.0 (2.0–13.0)	107
Mechanical ventilation—survivors (days, median (IQR))	2.0 (0.0–7.0)	49
Mechanical ventilation—non-survivors (days, median (IQR))	8.0 (3.0–16.0)	58
Renal replacement therapy (days, median (IQR))	8.0 (4.0–14.0)	107
Renal replacement therapy—survivors (days, median (IQR))	6.0 (3.0–14.0)	49
Renal replacement therapy—non-survivors (days, median (IQR))	9.0 (5.0–14.0)	58
Days on vasopressors (median (IQR))	5.0 (2.0–11.0)	106
Days on vasopressors—survivors (median (IQR))	2.5 (0.0–8.5)	48
Days on vasopressors—non-survivors (median (IQR))	6.0 (4.0–11.0)	58
Change in SOFA ^2^ score (T2-T1), mean (CI) ^5^	0.5 (−0.3 to 1.3)	73
Change in SOFA CNS ^3^ subscore	0.01 (−0.2 to 0.2)	92
Change in SOFA cardiovascular subscore	0.1 (−0.3 to 0.4)	95
Change in SOFA pulmonary subscore	0.04 (−0.2 to 0.2)	85
Change in SOFA coagulation subscore	0.6 (0.4 to 0.8)	90
Change in SOFA liver subscore	−0.1 (−0.3 to 0.1)	92
Change in SOFA renal subscore	0.1 (−0.3 to 0.5)	94
C-reactive protein at T2 (mg/L), mean ± SD ^6^; median (IQR)	48.5 ± 55.3; 32.6 (13.0–70.8)	69
Procalcitonin at T2 (ng/mL), mean ± SD; median (IQR)	5.9 ± 12.7; 1.4 (0.6–3.8)	49
Serum total bilirubin (mg/dL) at T2, median (IQR)	8.7 (4.8 to 17.6)	93
Platelets at T2 (G/L), mean ± SD	62.2 ± 59.4	91
Interleukine-6 at T2 (pg/mL), mean ± SD; median (IQR)	575.1 ± 1070.4; 237.7 (82.0–320.6)	7
Delta C-reactive protein (T2-T1) (mg/L), mean (CI)	−3.9 (−15.8 to 7.9)	60
Delta procalcitonin (T2-T1) (ng/mL), mean (CI)	0.8 (−3.5 to 5.1)	33
Delta total bilirubin (T2-T1) (mg/L), mean (CI)	−4.6 (−6.3 to −2.8)	92
Delta platelet (T2-T1) (G/L), mean (CI)	−34.1 (−45.5 to −22.8)	90
Assessment of change due to hemoadsorption		109
Improved, *n* (%)	75 (68.9)	
No change, *n* (%)	17 (15.6)	
Deteriorated, *n* (%)	5 (4.8)	
Not assessed, *n* (%)	12 (11.0)	

^1^ ICU: intensive care unit; ^2^ Sequential Organ Failure (SOFA) subscores range from 0 to 4 with an increasing score reflecting worsening of organ dysfunction. ^3^ CNS: central nervous system; ^4^ IQR: interquartile range; ^5^ CI: 95% confidence interval; ^6^ SD: standard deviation.

## Data Availability

The data presented in this study are available on request from the corresponding author. The data are not publicly available due to data protection regulations of the CytoSorb Registry.
